# Factors associated with an interruption in treatment of people living with HIV in USAID-supported states in Nigeria: a retrospective study from 2000–2020

**DOI:** 10.1186/s12889-021-12264-9

**Published:** 2021-11-30

**Authors:** Silviu Tomescu, Thomas Crompton, Jonathan Adebayo, Constance Wose Kinge, Francis Akpan, Marcus Rennick, Charles Chasela, Evans Ondura, Dauda Sulaiman Dauda, Pedro T. Pisa

**Affiliations:** 1 Right to Care, Centurion, South Africa; 2 Division of Epidemiology and Biostatistics, School of Public Health, Faculty of Health Sciences, University of the Witwatersrand, Johannesburg, South Africa; 3 The Palladium Group, Abuja, Nigeria; 4 Department of Human Nutrition and Dietetics, Faculty of Health Sciences, University of Pretoria, Pretoria, South Africa

**Keywords:** Retention, Loss to follow up (LTFU), Interruption in treatment (IIT), Multi-month dispensing (MMD), Antiretroviral treatment (ART), Nigeria, Continuity of care

## Abstract

**Background:**

Patient interruption of antiretroviral therapy (ART) continues to limit HIV programs’ progress toward epidemic control. Multiple factors have been associated with client interruption in treatment (IIT)— including age, gender, CD4 count, and education level. In this paper, we explore the factors associated with IIT in people living with HIV (PLHIV) in United States Agency for International Development (USAID)-supported facilities under the U.S. President’s Emergency Plan for AIDS Relief (PEPFAR) program in Nigeria.

**Methods:**

We conducted cross-sectional analyses on data obtained from Nigeria’s National Data Repository (NDR), representing a summarized record of 573 630 ART clients that received care at 484 PEPFAR/USAID-supported facilities in 16 states from 2000–2020. IIT was defined as no clinical contact for 28 days or more after the last expected clinical contact. Univariate and multivariate logistic regression models were computed to explore the factors associated with IIT. The variables included in the analysis were sex, age group, zone, facility level, regimen line, multi-month dispensing (MMD), and viral load category.

**Results:**

Of the 573 630 clients analysed in this study, 32% have been recorded as having interrupted treatment. Of the clients investigated, 66% were female (32% had interrupted treatment), 39% were aged 25–34 at their last ART pick-up date (with 32% of them interrupted treatment), 59% received care at secondary level facilities (37% interrupted treatment) and 38% were last receiving between three- to five-month MMD (with 10% of these interrupted treatment). Those less likely to interrupt ART were males (aOR = 0.91), clients on six-month MMD (aOR = 0.01), adults on 2^nd^ line regimen (aOR = 0.09), and paediatrics on salvage regimen (aOR = 0.02). Clients most likely to interrupt ART were located in the South West Zone (aOR = 1.99), received treatment at a tertiary level (aOR = 12.34) or secondary level facilities (aOR = 4.01), and had no viral load (VL) on record (aOR =10.02). Age group was not significantly associated with IIT.

**Conclusions:**

Sex, zone, facility level, regimen line, MMD, and VL were significantly associated with IIT. MMD of three months and longer (especially six months) had better retention on ART than those on shorter MMD. Not having a VL on record was associated with a considerable risk of IIT.

## Background

Globally there are an estimated 38 million PLHIV in 2019, with 2.9 million of those in the Western and Central African region [1]. The prevalence of HIV/AIDS in Nigeria is estimated at 1.4% of the population, with 1.8 million people living with HIV (PLHIV). The incidence rate decreased from 0.74 to 0.52 per 1 000 population from 2010 to 2019, ending with 100 000 new infections [2]. The UNAIDS 90-90-90 strategy aimed to have 90% of PLHIV know their status, and 90% of those to be retained on antiretroviral therapy (ART) (90% coverage) such that 90% of those are virally suppressed by 2020 [3]. More ambitious targets have also been set for 2030 to reach PEPFAR’s 95-95-95 goals for the care and treatment cascade [4]. Significant efforts have been deployed worldwide to make ART widely accessible to PLHIV following the “treat all” policy recommended by the World Health Organization (WHO) in 2016 [5]. In 2016, Nigeria started rolling out the “test and treat” policy [6]. The most recent estimate of ART coverage in Nigeria was 65% in 2019 [2], which is significantly below the coverage proportions targeted by UNAIDS and PEPFAR. There are currently an estimated 754 566 PLHIV in the 16 states supported by USAID under the PEPFAR program in Nigeria, with an estimated 467 567 PLHIV (62%) receiving ART [7]. Continuation of treatment is important because clients must achieve a suppressed viral load (VL), better client health outcomes, and epidemic control [8].

Client interruption in treatment was defined by PEPFAR in 2020 as no clinical contact for at least 28 days after the last clinical appointment or expected clinic visit. IIT is comparable to the loss to follow up (LTFU) concept as defined by WHO – with the distinction that LTFU is defined as no clinical contact for at least 90 days after the last expected clinical visit. In that regard, the PEPFAR definition of IIT supports more timely identification of clients interrupting treatment [9]. In this study we have adhered to the IIT concept as defined by PEPFAR . There are several factors associated with clients interrupting treatment, such as their age, gender, CD4 count, and geographical location [10, 11]. Potential risk factors identified in various studies include clients’ educational level, the lack of a telephone, and risky sexual behaviour [8, 12, 13]. Understanding the profile of clients at risk of IIT is invaluable in directing attention to them and minimizing this risk.

In this study, we explored the factors associated with treatment interruption by analysing de-identified data of more than 500 000 clients enrolled in ART programs in 16 USAID-supported sites in Nigeria between 2000 and 2020. The results of this two-decade large-cohort study can help ART program implementers understand the factors associated with IIT and plan for their mitigation to achieve better client retention. Similar studies have been carried out at different times in different areas of Nigeria. Out study contributes to the understanding of IIT in Nigeria [10, 14].

## Methods

### Study setting, design, and population

We conducted a retrospective cross-sectional analysis of routinely collected data from clients that received ART in 16 of Nigeria’s 36 states and the Federal Capital Territory (FCT) in Nigeria, based on a record review of client line-lists in electronic medical records (EMR) databases integrated into the National Data Repository (NDR) in Nigeria. The data comprised clients from 484 USAID-supported facilities across the 16 states that are currently supported by USAID. Data from clients enrolled in care from January 1, 2000, to December 8, 2020, were included in the study.

The 16 states in the study cohort were grouped into five different zones within Nigeria: North Central, North East, North West, South-South, and South West (see Table 1).

**Table 1:** Grouping of the 16 states into zones and the number of HIV clients per state

Zone	State
North Central	Kwara (9 110), Niger (31 817)
North East	Adamawa (49 084), Bauchi (29 670), Borno (21 989), Yobe (8 239)
North West	Jigawa (12 190), Kano (52 205), Kebbi (8 318), Sokoto (6 624)
South-South	Akwa Ibom (223 922), Bayelsa (13 971), Cross River (79 436), Edo (33 250)
South West	Lagos (73 495)

### Data source and management

The data were obtained from EMR databases integrated within the NDR. Data were collected using standardized national HIV data collection tools which record information on clients’ demographic, clinical, and treatment information at each encounter. The client information is stored both in paper-based and EMR systems. Client information is uploaded to the NDR through Extensible Markup Language (XML) files extract from the EMR.

**Fig. 1:**
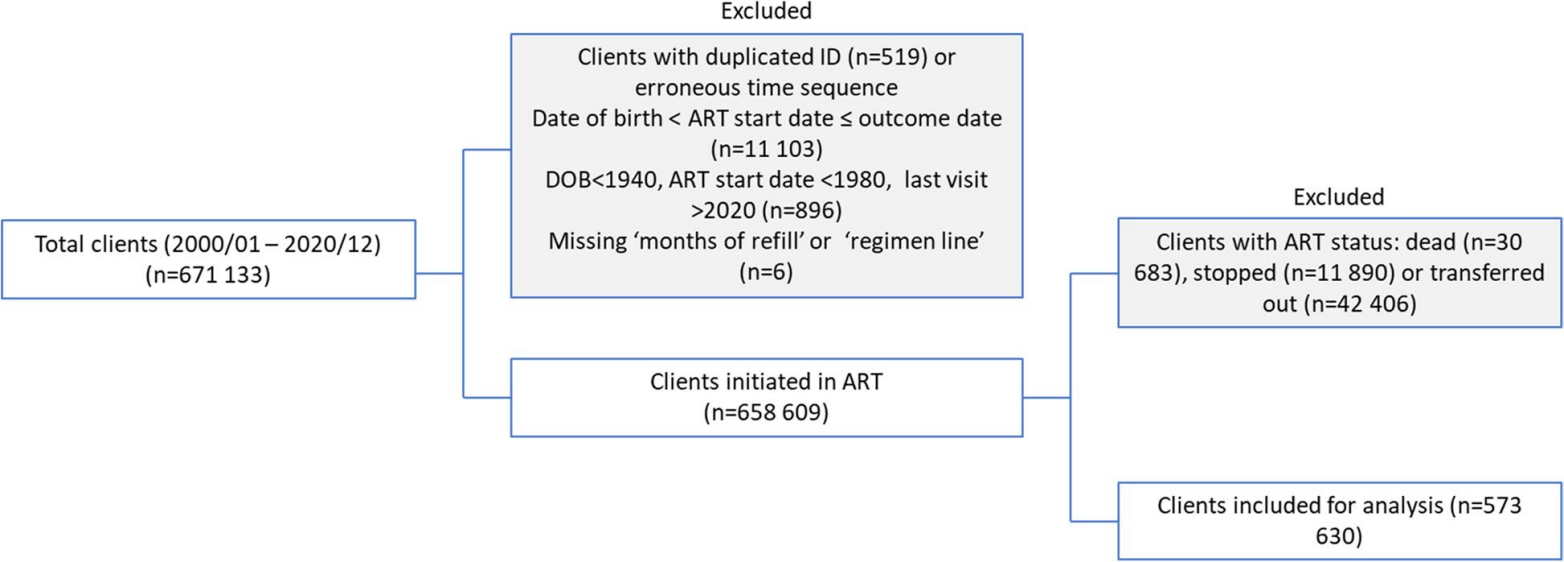
The data cleaning process, data excluded, and cohort subset analyzed

The original cohort data contained 671 133 records of clients. A unique identifier was created for each client concatenating the date of birth, sex, unique ID, and patient hospital number because the provided unique IDs and patient hospital numbers were missing for 218,656 records and could not be used. A completeness check was done for each variable and any misspelled outcomes were changed to reflect true values. Cleaning the dataset involved removal of duplicated unique identifiers, entries with a missing multi-month dispensing (MMD) value, and records that did not follow a logical chronological sequence―e.g., with ART start data occurring before the date of birth or occurring before the outcome date. Cases where the recorded date of birth was before 1940, the ART start date was before 1980, or the last ART pickup date was after 2020 were excluded (Fig. 1).

We removed 519 duplicate entries from the cohort data set, as well as three entries with a missing MMD value and 11, 03 records that did not follow a logical chronological sequence―with the date of birth occurring before the ART start date, and/or occurring before the outcome date. An additional 896 records were excluded in cases when the date of birth occurred before 1940, the ART start date was before 1980, or when the last ART pickup date was after 2020 (see Fig. 1). From the 658 609 retained complete client records, 30 683 (5%) were recorded as dead; 11 890 (2%) were recorded as having stopped ART, and 42 406 (6%) were recorded as transferred out. Those records were removed before logistic regression analysis. After data cleaning, all client records were complete ( there were no missing variables for each client record).

The outcome date for treatment interruption was calculated as the last pick-up date plus the number of days of ART dispensed plus 28 days, in line with the PEPFAR guidelines [9]. For the analysis, clients with the recorded outcome as active, active-restart, and active-transfer-in were treated as active. By contrast, clients recorded as dead, stopped, or transferred out were excluded from the analysis; some cases were excluded if there was insufficient information to categorize the outcome of those clients as either active or interrupting treatment (Fig. 1). This was done to ensure that comparisons of IIT risks were made to currently active clients.

### Computed variables explored as predictors of IIT

Facility levels were assigned according to the Nigerian Health Facility Registry [15]. That is, the 484 facilities were grouped into primary level (236), secondary level (228), and tertiary level (20). In Nigeria, primary levels facilities provide the minimum level of care to clients and operate at the Local Government Area. Secondary level facilities operate at State level and provide more specialised care to clients, while tertiary level facilities operate at Federal level and provide the most advanced level of care to clients [16].

The current ART regimen line described the regimen line which the client was on at their last recorded visit. The VL variable was categorised into three levels: “suppressed” – clients whose last VL on record was below 1,000 copies per millilitre (c/ml); “unsuppressed” – clients whose last VL on record was at least 1,000 (c/ml); and “not recorded” – clients who did not have a VL reading on record [6].

### Statistical analysis

The association of each variable with treatment interruption was tested using Pearson’s chi-squared test. Both crude and adjusted logistic regression models were computed to explore associations with IIT. Backward elimination was used to determine the best set of variables to include using the Akaike information criterion (AIC) statistic to evaluate model performance. All regressions were computed using two models. The first model was a crude, univariate logistic regression and reported the unadjusted odds ratio (OR); the second model adjusted for all covariates and the adjusted odds ratio (aOR) was reported. Variables included in the analysis were sex, age group, zone, facility level, regimen line, MMD, and VL category. Statistical significance was defined using a two-tailed *p*-value <0.000001 [17]. Multicollinearity tests were done using the variance inflation factor test. All data were analysed using the software R for Statistical Computing v4.0.2 [18].

## Results

### Cohort characteristics

Of the 573,630 clients included in our analysis, 183,046 (32%) since 2000 were classified as having interrupted treatment; the rest were considered active. Two-thirds of the clients initiated into ART were females and approximately one-third of males and females had interrupted treatment since the study period began. The largest age groups at ART start were 25- to 34- and 35- to 44-year-olds, accounting for 39 and 25% of the cohort, respectively. The largest age groups on ART at the client outcome date were still 25- to 34- and 35- to 44-year-olds; however, the proportion of the groups had shifted to 32 and 30%, respectively. Overall, we observed a proportional shift from younger age groups (below age 25) at ART start to more mature age groups at outcome date (Table 2). The South-South Zone of Nigeria which contains Akwa Ibom, the state with the highest HIV prevalence in the country, had the largest representation within the cohort in the study, accounting for 53% of the clients initiated into ART, followed distantly by the North East, which contributed 16% of the cohort. The South West zone of Nigeria had the highest proportion of clients interrupting treatment (49%). Secondary and primary level health facilities accounted for 59 and 25% of the clients registered to facilities, respectively. However, tertiary and secondary level facilities accounted for the highest number of clients interrupting treatment, with 41 and 37% of the clients enrolled in those facilities, respectively (see Table 2).

**Table 2:** Patient characteristics stratified by treatment interruption

Factors	Treatment interruptions			
	No (%)	Yes (%)	Total (%)	***p***-value
Sex				
Female	258 926 (68)	119 540 (32)	378 466 (66)	<0.000001
Male	131 658 (67)	63 506 (33)	195 164 (34)	
Age group at ART start				
0–14	18 059 (64)	10 126 (36)	28 185 (5)	<0.000001
15–24	66 479 (65)	35 543 (35)	102 022 (18)	
25–34	152 473 (68)	71 631 (32)	224 104 (39)	
35–44	100 090 (70)	42 068 (30)	142 158 (25)	
45–59	48 021 (70)	20 715 (30)	68 736 (12)	
60+	5 462 (65)	2 963 (35)	8 425 (1)	
Age group				
0–14	13 900 (60)	9 080 (40)	22 980 (4)	<0.000001
15–24	38 231 (60)	25 956 (40)	64 187 (11)	
25–34	120 558 (65)	65 931 (35)	186 489 (33)	
35–44	123 625 (71)	49 653 (29)	173 278 (30)	
45–59	79 890 (74)	27 592 (26)	107 482 (19)	
60+	14 380 (75)	4 834 (25)	19 214 (3)	
Zone				
South-South	217 601 (71)	87 480 (29)	305 081 (53)	<0.000001
North Central	32 701 (86)	5 211 (14)	37 912 (7)	
North East	58 339 (64)	33 131 (36)	91 470 (16)	
North West	49 162 (65)	26 342 (35)	75 504 (13)	
South West	32 781 (51)	30 882 (49)	63 663 (11)	
Facility level				
Primary	125 158 (86)	20 347 (14)	145 505 (25)	<0.000001
Secondary	213 505 (63)	126 249 (37)	339 754 (59)	
Tertiary	51 921 (59)	36 450 (41)	88 371 (15)	
Regimen line				
Adult 1^st^ Line	369 290 (68)	173 205 (32)	542 495 (95)	<0.000001
Adult 2^nd^ Line	9 770 (75)	3 198 (25)	12 968 (2)	
Adult 3^rd^ Line	11 (100)	0 (0)	11 (0)	
Peds 1^st^ Line	10 985 (63)	6 512 (37)	17 497 (3)	
Peds 2^nd^ Line	509 (80)	128 (20)	637 (0)	
Salvage	19 (86)	3 (14)	22 (0)	
MMD				
1	11 842 (12)	90 756 (88)	102 598 (18)	<0.000001
2	15 395 (18)	68 913 (82)	84 308 (15)	
3-5	199 678 (90)	21 098 (10)	220 776 (38)	
6	163 669 (99)	2 279 (1)	165 948 (29)	
VL				
0 – Suppressed	272 968 (94)	18 237 (6)	291 205 (51)	<0.000001
Not recorded	90 281 (37)	156 449 (63)	246 730 (43)	
Unsuppressed	27 335 (77)	8 360 (23)	35 695 (6)	

The most common ART regimen line was the adult first-line regimen, having been prescribed to 95% of the cohort at their last visit date; 32% of these clients had interrupted treatment. Only 2% of clients were transitioned to second-line treatment, and 25% of these interrupted treatments.

Multi-month dispensing of three- to five-month and six-month ART regimens were the most common options, having been issued to 38 and 29% of clients respectively, at their last visit date. Most clients were issued one-month and two-month ART regimens; 90,756 (88%) and 68,913 (82%), respectively, had interrupted treatment; together accounting for 87% of all the clients interrupting treatment (50 and 37%, respectively). More than half of the cohort in the study, 291,205 (51%) had a suppressed VL, and only 6% of those had interrupted ART; 35,695 (6%) of clients in the cohort were recorded to have an unsuppressed VL, and 23% of those interrupted ART. The remaining 246,730 (43%) of clients did not have a recorded VL; 63% of those had interrupted treatment.

### Factors associated with treatment interruption

Results from the multivariate analysis (Table 3) showed that male clients were 9% (95% CI 0.89-0.93, *p*<0.000001) less likely to interrupt treatment; the adjusted ORs for all age groups were not statistically different from each other considering the sample size was in the hundreds of thousands (see Table 9 in reference [17] for statistical significance guidelines), indicating the age has little effect on treatment interruption. The North East (aOR = 1.36, 95% CI 1.32 - 1.4, *p*<0.000001) and South West zones (aOR = 1.99, 95% CI 1.92-2.06, *p*<0.000001) were associated with higher odds of treatment interruption, whereas the North Central zone had the lowest odds (aOR = 0.4, 95% CI 0.38-0.42, *p*<0.000001) compared to the South-South zone. Clients receiving treatment at secondary and tertiary level healthcare facilities accounted for 64% of the study population and both were associated with clients more likely to interrupt treatment compared to clients receiving treatment at primary level facilities, adjusted ORs of 4.01 (95% CI 3.9-4.13, *p*<0.000001) and 12.34 (95% CI 11.87-12.82, *p*<0.000001) respectively. Clients that were switched to second-line regimens (aOR = 0.09, 95% CI 0.09 - 0.1, *p*<0.000001) had lower adjusted odds of interrupting ART when compared with their counterparts on first-line regimens. Clients on MMD had the largest impact on treatment interruptions of all variables analysed, with clients on six-month MMD 99 % (aOR = 0.01, 95% CI 0-0.01, *p*<0.000001) less likely to interrupt treatment than those on one-month prescriptions. Clients that had no VL on record were 10.02 times (95% CI 9.76-10.28, *p*<0.000001) more likely to interrupt ART compared to clients with suppressed VL. Backward and forward elimination of factors for the multivariate logistic regression model identified that the age group had the least improvement contribution to the model, reducing the Akaike Information Criterion (AIC) from 251 092.9 to 251 075.5, however, because this a principal demographic indicator and the model was not worsened, the age group variable was retained.

**Table 3:** Factors associated with treatment interruption

Factors	Univariate		Multivariate	
	OR [95% CI]	***p*** (<0.000001)	OR [95% CI]	***p*** (<0.000001)
Sex				
Female	1 [ref]	0	1 [ref]	0
Male	1.04 [1.03 - 1.06]	<0.000001	0.91 [0.89 - 0.93]	<0.000001
Age group				
0-14	1 [ref]	<0.000001	1 [ref]	0
15-24	1.04 [1.01 - 1.07]	0.014073	1.08 [0.97 - 1.19]	0.16165
25-34	0.84 [0.81 - 0.86]	<0.000001	1.19 [1.08 - 1.32]	0.000539
35-44	0.61 [0.6 - 0.63]	<0.000001	1.13 [1.02 - 1.25]	0.0185935
45-59	0.53 [0.51 - 0.54]	<0.000001	1.08 [0.97 - 1.19]	0.147981
60+	0.51 [0.49 - 0.54]	<0.000001	1.22 [1.08 - 1.36]	0.000822
Zone				
South-South	1 [ref]	0	1 [ref]	0
North Central	0.4 [0.38 - 0.41]	<0.000001	0.4 [0.38 - 0.42]	<0.000001
North East	1.41 [1.39 - 1.43]	<0.000001	1.36 [1.32 - 1.4]	<0.000001
North West	1.33 [1.31 - 1.36]	<0.000001	0.83 [0.8 - 0.85]	<0.000001
South West	2.34 [2.3 - 2.38]	<0.000001	1.99 [1.92 – 2.06]	0
Facility level				
Primary level	1 [ref]	0	1 [ref]	0
Secondary level	3.64 [3.58 - 3.7]	<0.000001	4.01 [3.9 - 4.13]	<0.000001
Tertiary level	4.32 [4.23 - 4.41]	<0.000001	12.34 [11.87 - 12.82]	<0.000001
Regimen line				
Adult first line	1 [ref]	0	1 [ref]	0
Adult.2nd.Line	0.7 [0.67 - 0.73]	<0.000001	0.09 [0.09 - 0.1]	0
Adult.3rd.Line	0 [0 - 5.91e+14]	0.686815	0 [0 – 4.45e+20]	0.702157
Peds.1st.Line	1.26 [1.23 - 1.3]	<0.000001	0.35 [0.32 - 0.39]	<0.000001
Peds.2nd.Line	0.54 [0.44 - 0.65]	<0.000001	0.08 [0.06 - 0.1]	<0.000001
Salvage	0.34 [0.1 - 1.14]	0.079702	0.02 [0 - 0.06]	<0.000001
MMD				
1	1 [ref]	0	1 [ref]	0
2	0.7 [0.67 - 0.73]	<0.000001	0.78 [0.76 - 0.81]	<0.000001
3-5	0.1 [0.09 - 0.1]	<0.000001	0.02 [0.02 - 0.02]	<0.000001
6	0.04 [0.03 - 0.04]	<0.000001	0.01 [0 - 0.01]	<0.000001
VL				
Suppressed	1 [ref]	0	1 [ref]	0
Not recorded	7.1 [6.89 - 7.32]	<0.000001	10.02 [9.76 – 10.28]	<0.000001
Unsuppressed	1.99 [1.9 - 2.09]	<0.000001	2.23 [2.14 - 2.33]	<0.000001

## Discussion

In this study, we investigated risk factors that were associated with treatment interruptions in a large cohort of clients receiving ART in Nigeria. Our results show that MMD of more than three months was highly associated with reduced treatment interruption, while six months of MMD had the best retention.

These results are similar to findings in Malawi and Zambia where six-month regimens of MMD were ideal for retention and shorter MMD were less protective [19]. Other studies have also found that one- and two-month ART regimens have greater odds of treatment interruption. More specifically, 49.4 and 26.7% of interruption in treatment associated with one- and two-month ART regimen, respectively, compared with our study which has 78% of interruption in treatment associated with a two-month ART regimen [20, 21]. Findings from a similar study indicated 49% of clients on one-month regimens had interrupted treatment [10], while that proportion was 50% in this cohort. The higher proportion of ART interruptions associated with shorter dispensing durations can be explained by the requirement for frequent visits for the client that was associated with a higher rate of ART interruption [22].

Virally suppressed clients were shown to have a lower risk of ART interruptions compared to clients with an unsuppressed VL, while clients with no VL record had the highest risk. The higher level of interruption is logically associated with virally unsuppressed clients, likely due to poor adherence. Also, the high treatment interruption levels associated with clients without a recorded VL may be indicative of the poor quality of clinical care, which was associated with ART interruption [22]. Increasing the frequency of VL testing and making it accessible to more clients could encourage client involvement in ART programs. Further, receiving treatment at a secondary or tertiary level facility increased the likelihood of treatment interruption compared to clients receiving treatment at primary level facilities. A potential reason could be the higher client volumes at these sites and possibly busier, metropolitan schedules. Nevertheless, with higher client turnout in an urban settlement, chances are that there is increased workload for health workers and this may result in a shorter duration of consultations and poor quality of service. Similarly, clients at facilities in the North East and South West states were associated with higher rates of IIT. Potential reasons for the high rates of IIT are that the North East zone of Nigeria has been experiencing security and other challenges which make travel and attending a clinic more difficult, whereas the South West zone contains Lagos which although it is has the smallest area compared to other Nigerian states, it also has the highest urban population which was associated with higher rates of LTFU [23–25]. Age and sex were shown to not have a considerable association with treatment interruption when compared and adjusted for the other variables in our study, results similar to those found in other studies which did not find a stronger association of older age with IIT [26]. Other studies which did associate an elder age group with IIT explained the occurrence as a possible reflection of inaccurate classification of IIT after the clients’ death [10]. Earlier studies have shown a higher likelihood for males to interrupt treatment and recommended male-targeted interventions [10, 14]; this may have resulted in improved odds for males to adhere to treatment.

The 32% treatment interruption proportion is similar to the 33% client interruption rate in Nigeria following a cohort from 2004–2017 [10]. The proportion of treatment interruption may vary between studies depending on the level at which the study is carried out. Studies at the facility level in Nigeria identified that 56% of clients interrupted ART at the Lagos University Teaching Hospital [27], while an earlier study carried out on randomly sampled data (2004–2012) from 35 sites in Nigeria identified 75% of clients interrupting treatment [28]. Transferring to another program may be one of the principal reasons for failure to return to the same clinic [29]. For that reason, studies focused above the facility level may be better at identifying interruptions by accounting for transfers between programs.

Our study was based on a large cohort of over half a million clients receiving ART over 20 years; this suggests that the sample size is robust and is representative of the sixteen states included in this study which are supported by PEPFAR/USAID, while generalizability to other Nigerian states is limited. Another key limitation of our study is the availability of client information only at their last visit date. We do not know when a client transitioned from one-month dispensing to MMD and cannot compare rates of ART interruption longitudinally. A further limitation is that the six-month MMD program has been in place only since late 2019, that period may not be long enough to adequately monitor the effects of this switch on treatment interruption. Categorizing active-restart and active transfer-in clients as active clients may be a limitation of the study that could introduce bias in the results despite having an active status at the last clinic visit. Noteworthy, an active-restart status is recorded as such until the end of the reporting period, thereafter, clients are classified as active. While active-transfer-in clients are clients that have transferred in from a facility that was not supported by PEPFAR [9].

For further analyses, we suggest comparing treatment interruption rates longitudinally to account for changes in MMD, regimen lines, and the regimen itself. Allowing more time for the six-month MMD program to be evaluated would lead to more conclusive results on whether this has led to a reduction in treatment interruption in Nigeria.

## Conclusions

Sex, zone, facility level, regimen line, MMD, and VL were significantly associated with IIT. Multi-month dispensing of three months and longer (especially six months) was associated with better treatment continuity than those on shorter courses of MMD. Not having a VL on record was associated with a considerable risk of IIT.

## Data Availability

The datasets generated and/or analysed during the current study are not publicly available due the data being representative of a cohort of ART clients in Nigeria whose personal records are protected by relevant national data protection regulations but are available from the corresponding author on reasonable request.
